# T Cell Receptor Excision Circle (TREC) Monitoring after Allogeneic Stem Cell Transplantation; a Predictive Marker for Complications and Clinical Outcome

**DOI:** 10.3390/ijms17101705

**Published:** 2016-10-11

**Authors:** Ahmed Gaballa, Mikael Sundin, Arwen Stikvoort, Muhamed Abumaree, Mehmet Uzunel, Darius Sairafi, Michael Uhlin

**Affiliations:** 1 Department of Oncology and Pathology, Karolinska Institutet, SE-141 86 Stockholm, Sweden; Ahmed.Gaballa@ki.se (A.G.); Arwen.Stikvoort@ki.se (A.S.); Darius.Sairafi@ki.se (D.S.); 2 Division of Pediatrics, Department of Clinical Science, Intervention and Technology, Karolinska Institutet, SE-141 86 Stockholm, Sweden; Mikael.Sundin@ki.se; 3 Pediatric Blood Disorders, Immunodeficiency and Stem Cell Transplantation, Astrid Lindgren Children’s Hospital, Karolinska University Hospital, SE-141 86 Stockholm, Sweden; 4 Stem Cells and Regenerative Medicine Department, King Abdullah International Medical Research Center (KAIMRC), King Saud bin Abdulaziz University for Health Sciences, King Abdulaziz Medical City, Ministry of National Guard Health Affairs, KSA-11461 Riyadh, Saudi Arabia; AbumareeM@ksau-hs.edu.sa; 5 Department of Clinical Immunology and Transfusion Medicine, Karolinska University Hospital, SE-141 86 Stockholm, Sweden; Mehmet.Uzunel@ki.se

**Keywords:** TREC, transplantation, immune reconstitution

## Abstract

Allogeneic hematopoietic stem cell transplantation (HSCT) is a well-established treatment modality for a variety of malignant diseases as well as for inborn errors of the metabolism or immune system. Regardless of disease origin, good clinical effects are dependent on proper immune reconstitution. T cells are responsible for both the beneficial graft-versus-leukemia (GVL) effect against malignant cells and protection against infections. The immune recovery of T cells relies initially on peripheral expansion of mature cells from the graft and later on the differentiation and maturation from donor-derived hematopoietic stem cells. The formation of new T cells occurs in the thymus and as a byproduct, T cell receptor excision circles (TRECs) are released upon rearrangement of the T cell receptor. Detection of TRECs by PCR is a reliable method for estimating the amount of newly formed T cells in the circulation and, indirectly, for estimating thymic function. Here, we discuss the role of TREC analysis in the prediction of clinical outcome after allogeneic HSCT. Due to the pivotal role of T cell reconstitution we propose that TREC analysis should be included as a key indicator in the post-HSCT follow-up.

## 1. Allogeneic Stem Cell Transplantation

Over the past five decades, progress within the field of allogeneic hematopoietic stem cell transplantation (HSCT) has been astonishing, making it one of current medicine’s fastest expanding disciplines. Advances have markedly reduced risks, improved outcome, and widened the indications for the procedure. HSCT is currently a valid second or third line option for life-threatening conditions when no alternative treatments are available. The difficulty in foreseeing certain complications post-HSCT, such as relapse and infectious complications, is one of the main problems.

### 1.1. Indications for Hematopoietic Stem Cell Transplantation (HSCT)

Initially, HSCT was restricted to patients with acute leukemia, severe aplastic anemia (SAA), or severe combined immunodeficiency (SCID) [1,2]. Over the years indications for treatment have widened to chronic leukemias, lymphomas, multiple myeloma, myelodysplastic syndromes, and diverse forms of inherited metabolic, hematologic and immunologic disorders. Additionally, HSCT has been evaluated—with encouraging results—as a treatment for other diseases not conventionally considered for transplant, for instance renal cell carcinoma, sickle cell anemia, beta thalassemia major, and some autoimmune disorders [3,4,5,6,7].

Clinical HSCT is a quickly changing field with new methods, guidelines and treatment modalities frequently presented into routine practice. The European group for Blood and Marrow Transplantation (EBMT) and its American equivalent, Center for International Blood and Marrow Transplant Research (CIBMTR), frequently update guidelines and recommendations regarding the current practice of and indications for HSCT [8,9].

### 1.2. Conditioning Therapy

A preparative treatment (conditioning) starts the HSCT procedure. It aims to prevent graft rejection and, in malignancies, to reduce the relapse risk. Initially, total body irradiation (TBI) and high dose cyclophosphamide (Cy) were used as separate conditioning regimens and were later combined for better effect [10,11]. Results were promising; more than half of the initial patients remained disease-free five years after transplant [12]. Introducing the alkylating agent busulfan (Bu) in later transplants offered an alternative to the logistically more demanding TBI-based regimens [13,14].

As early as the 1950s it was discovered that HSCT provided an additional anti-leukemic effect to that delivered by the myeloablative conditioning alone [15,16]. It eventually became clear that sustained disease remission after HSCT was highly dependent on a continued reaction between the allogeneic immune system and the malignant recipient cells [17,18,19]. Based on these findings, new conditioning regimens were developed [20,21,22,23]. These new non-myeloablative or reduced-intensity conditioning (RIC) regimens were associated with a significant reduction of transplant-related mortality (TRM). This development also made HSCT available for new cohorts of patients for whom the treatment had previously not been considered a safe option, i.e. older patients or those with co-morbidity.

Conditioning regimens are, in some instances, combined with the use of inhibiting monoclonal or polyclonal antibodies against T cells [24]. The main effect of the anti-T cell serotherapy is to prevent rejection by a reducing patient T cell numbers. However, anti-T cell serotherapy may also reduce the risk for graft-versus-host-disease (GVHD) through a delayed suppressive effect on donor T cells.

### 1.3. Infectious Complications

The first months post-HSCT are characterized by profound immunodeficiency, in which the patients are at risk of opportunistic infections. Susceptibility to microbial pathogens decreases gradually as the immune system regains its functionality. Different periods can be distinguished based on the incidence of specific infections after HSCT. The predominance of certain pathogens in each period is a reflection of the type of cell subset deficiency post-HSCT.

Not only immune reconstitution is linked to the occurrence of infections post-HSCT. There is also an association between acute GVHD and an increased predisposition to infections. This is thought to be a consequence of the immune modulatory effect of the ongoing systemic inflammation. However, the disturbance of epithelial barriers during conditioning is also a contributing factor [25,26]. In addition, the immunosuppressive drugs used to treat GVHD impair the immune system’s ability to combat infections.

### 1.4. T Cells—The Double Edged Sword

GVHD, i.e., an unwanted immunologic reaction of donor lymphocytes against patient tissue, remains one of the major obstacles post-HSCT, as it causes significant morbidity and mortality. GVHD is seen in both an acute and chronic form, with individual symptoms and diverse pathophysiological mechanisms, which are not all well understood [27].

Most patients undergoing HSCT receive continuous immunosuppressive treatment during the first 3–6 months post-HSCT to prevent an allogeneic response against healthy tissue. Methotrexate (MTX) and/or cyclosporine A (CsA) is mostly used. All currently available GVHD prophylaxis options inhibit T cell reactivity [28,29,30].

Donor T cells can be inactivated for a long period through administration of neutralizing anti-T cell antibodies, either ex vivo or in vivo. These approaches are collectively termed T cell depletion. While they have the potential to eliminate the risk for GVHD, there is also a significantly increased risk of relapse.

In parallel with the development of more effective immunosuppressive protocols, advances in human leukocyte antigen (HLA)-typing have significantly contributed to reducing the incidence and severity of GVHD, increasing the rates of engraftment and improving overall survival (OS) [31,32,33].

The fundamental treatment option for acute GVHD is still systemic administration of corticosteroids in addition to standard immunosuppression protocols.

The effectiveness of HSCT against malignant disorders is dependent on the immunological response between donor-derived lymphocytes and neoplastic cells. This is referred to as the graft-versus-leukemia (GVL) or graft-versus-tumor (GVT) reaction, and explains why HSCT, despite the risk of severe complications, still offers a survival advantage for patients with late-stage malignant disorders when compared to other treatments. Several observations have led to the general recognition of the importance of this phenomenon.

In the clinical setting, it was noted that patients who developed GVHD after HSCT had a significantly lower risk of relapse than those who did not show any symptoms of GVHD [18,19,34]. Analogously, it was shown that discontinuation of immunosuppression, with the resulting occurrence of GVHD, could be used to re-establish remission in the case of relapsed leukemia after HSCT [35,36,37].

The vital role of allo-reactive T cells in GVT reaction and GVHD is illustrated by the fact that both processes are virtually absent in transplantations with grafts fully depleted of T cells [38]. In addition, they seem to depend strongly upon some degree of histo-incompatibility between donor and recipient. There is also data suggesting that NK cells may contribute to the GVT reaction. This seems to be more pronounced against malignant cells of the myeloid lineage through inhibitory and activating NK cell receptors [39,40,41].

The GVT effect is in many cases associated with ongoing GVHD, presumably because of common elements in the mechanism of action [18,19,38,42]. However, this seems only to be the case if the GVH reaction is initiated against host-specific antigens expressed by malignant cells as well as healthy host cells [43,44,45]. It is also reasonable to conclude that these antigens often consist of minor histocompatibility antigens (MiHA) rather than HLA, as the GVT effect is present in transplantations with HLA-identical siblings and there does not seem to be a significant difference in the relapse rate between HSCT with HLA mismatched (MM) and fully matched donors [46]. Moreover, some antigens are only expressed on tumor cells. These tumor-specific or tumor-associated antigens do not seem to be able to autonomously elicit an allogeneic reaction but may contribute to the GVL effect once an immune response against MiHA has been established [47,48,49,50,51].

Many investigators believe that the GVT effect may be separated from GVHD and this is currently the subject of active research.

## 2. T Cell Reconstitution after HSCT and T Cell Receptor Excision Circles (TRECs)

Unlike innate immunity, which recovers early following HSCT, reconstitution of the adaptive immunity is a prolonged, if ever complete, process [52]. Early T cell recovery is associated with a better clinical outcome [53,54,55]. T cell reconstitution following HSCT depends on two major pathways. Firstly, the thymic-independent pathway in which peripheral clonal expansion, within the recipient, of donor-derived mature T cells from the graft takes place. This provides transient protection early post-HSCT as these cells respond and proliferate quickly when challenged with previously encountered pathogens [56]. However, these cells are of limited importance since they have a restricted repertoire and are potentially alloreactive. The second pathway, the thymic-dependent pathway, is a long-term process of de novo generation of naïve T cells differentiated in the recipient thymus [54,55,57,58]. These two reconstituted T cell populations develop in completely different antigenic environments (donor vs. recipient). The donor selected T cells are more likely to be alloreactive, whereas the recipient selected T cells are not. Studies have shown that GVHD is most likely caused by the alloreactive donor selected T cells that were co transplanted with the graft [55].

The thymus plays a fundamental role in the recovery of the T cell pool after HSCT and therefore also for long-term immune protection and tolerance. The extent to which the thymus can contribute to T cell reconstitution varies depending on several transplant related factors [52]. Monitoring recent thymic emigrants (RTE) could be of value in HSCT as it would help determine factors affecting immune recovery. Additionally, it would help to gain new insights into the contribution of the thymus in maintenance of the peripheral T cell compartment, eventually leading to establishing new strategies aimed at improving T cell reconstitution. A quantitative, precise and noninvasive biomarker for estimating thymic output was first described by Douek et al. [59]. It is based on measurement of the extrachromosomal DNA excision circles that are generated following T cell receptor (TCR) gene rearrangement in the thymus, the so called T cell receptor excision circles (TRECs). These stable DNA circles do not replicate during mitosis, instead they are diluted with each cellular division and can persist for a long time in mature T cells [55,58,60,61].

### 2.1. Methods to Detect TREC

The process of TCR rearrangement has been reported in all TCR genes; *TCRD, TCRG, TCRB* and *TCRA*. Following rearrangement in each gene, one or more TRECs are generated resulting in many different TRECs [62]. A good candidate TREC must be detectable in peripheral blood and not be affected by the different rearrangement possibilities. *TCRD* and *TCRG* genes undergo rearrangements in very early stages, therefore their TRECs are extensively diluted before they enter the peripheral blood. Similarly, TRECs derived from *TCRB* rearrangements undergo dilution in the thymus so their concentration in the periphery is very low compared to other TRECs generated from later rearrangements. Rearrangement of *TCRA* requires the deletion of the *TCRD* gene that is interspersed with *TCRA* along the same chromosomal location 14q11. This deletion occurs late, making the generated TREC less diluted by thymocyte expansion. Furthermore, it has been shown that approximately 70% of these *TCRD* deletion rearrangements result in a δRec-ΨJα signal joint and coding joint [59,60,62]. The δRec-ΨJα coding joint is found in the final rearrangement of Vα-Jα signal TREC but might also be found on one allele of genomic DNA. Since there is no possibility of distinguishing between them, the δRec-ΨJα signal joint TREC (sjTREC) is the optimal target for measurement in clinical setting [60,63].

As TREC is a DNA byproduct, the methods developed for its detection are PCR-based. Accordingly, different methods have been used following the advances in the field of molecular diagnostics. As in any PCR technique, contamination of reagents, samples and equipment are the most limiting factor. The earliest method described by Douek et al. [59] was a semi-quantitative PCR assay in which TREC count was determined by separating PCR products on polyacrylamide gels followed by measuring band intensity with a phospho-imager. Real time PCR was then introduced as it carries major advantages compared to conventional PCR. For instance, it permits monitoring the progression of the PCR reaction in each cycle; no radioactive reagents are used, and it is less time-consuming. Different methodologies have been utilized based on signaling systems. An approach using a molecular beacon in combination with real-time PCR was introduced for the detection of TREC by Zhang et al. [64]. The molecular beacon was included in the PCR reaction to serve as a real-time detector for the amplification. Alternatively, quantification of TREC using hybridization probes has been described [65,66]. Another approach based on the binding of SYBR-Green dye to the double stranded PCR products has been discussed. Although this method is cheaper, it is less specific as the binding of SYBR Green to DNA is sequence-independent. Therefore, it is essential to make sure that primer design and concentration are maximally optimized [67]. Alternatively, PCR-ELIZA assay has been described [62]. So far, the gold-standard technique is real-time PCR based on TaqMan site-specific probes containing a quencher and a reporter dye [53,55,57,61,68].

It is noteworthy that published results of TRECs show great variation; this is most likely explained by the variability in method design. For instance, some studies have used the absolute quantification of TREC, while in other experiments relative quantification by the delta-CT method has been used [69,70]. Moreover, quantification of TREC has been performed in different subpopulations. For instance, some investigators counted TREC in purified CD3^+^, CD4^+^ or CD8+ T cells [53,59,61,71]. In addition, TREC results have been expressed in different ways such as TREC per cell count [55], TREC per mL or µL of blood [53,54,72] or even TREC per µg of DNA [58,66].

Importantly, TREC results should be carefully interpreted to avoid erroneous conclusions, particularly since sjTREC levels are also influenced by other factors such as longevity of naïve T cells, peripheral expansion or apoptosis of T cells and intracellular degradation [54,66,71,73]. In order to overcome this limitation, Dion et al. [74] developed a novel approach that allows diminishing the effect of peripheral expansion. In their technique, sjTREC and βTREC were measured simultaneously and the sjTREC/βTREC ratio was determined. Although this methodology is more informative, it is laborious, expensive and time-consuming. Later on, several investigators developed simplified methods to determine sjTREC/βTREC ratio [71,75].

### 2.2. Alternative Methods for Detecting Thymic Output

Unfortunately, the study of T cell recovery in humans is limited by the fact that peripheral blood is the only accessible compartment for routine measurements [56,76]. Furthermore, indirect methods that have been commonly used to evaluate thymic output, such as phenotyping of the naïve T cells and chest-computed tomography (CT), are not reliable. Naïve T cells do not accurately measure RTE as they can have a longer life span, may proliferate or may convert into memory T cells. In addition, certain memory cells can acquire naïve-like phenotypes [56,58,66]. Similarly, radiographic imaging has considerable limitations as it provides a semi quantitative estimate of thymic output based on evaluation of thymic size and cellularity that does not accurately correlate with thymic output [53,66].

The diversity of the T cell receptor repertoire after HSCT depends mainly on the ability of the thymus to export new naïve T cells. Thus, measuring TCR diversity by means of spectratyping or sequencing of CDR3 of the β chain could be used as an alternative method to indirectly assess thymic output [77,78].

The fact that TREC data are hampered by several clinical and experimental factors and do not necessarily reflect a dynamic measurement of thymic output has encouraged many investigators to search for novel phenotypic markers that could be used as surrogates for assessing RTE. Two important markers that have been described are CD31 (PECAM-1) and protein tyrosine kinase 7 (PTK7). CD31 is a 130-kDa transmembrane glycoprotein that is expressed by a variety of cell types. Based on this surface molecule, CD4^+^ naïve T cells could be subdivided into CD31^+thymic^ and CD31^−central^ cells. It has been shown that CD31^+thymic^ naïve CD4^+^ T cell subsets contain higher sjTREC than CD31^−central^ naïve CD4^+^ T cell subsets. Furthermore, their frequency in peripheral blood decreases with age and displays a broader TCR repertoire. In addition, telomere length and telomerase activity were higher in CD31^+^ compared to CD31^−^ naïve T cells, indicating a lower replicative history. Therefore, CD31^+thymic^ naïve CD4^+^ T cells could be a suitable candidate to assess thymic output, however, it should be noted that not all CD31^+^ cells are necessarily RTE [71,79,80,81]. Alternatively, studies have shown that PTK7^+^ naïve T cells contain higher levels of TRECs. Furthermore, PTK7^+^ cells produce less IL-2 and IFN gamma compared to the PTK7^−^ subset suggesting that this novel marker can identify RTE [82] (Table 1).

### 2.3. Clinical Applications of TREC

TREC quantification has been used to assess thymic output in several clinical settings where T lymphocytes are essential for clinical evaluation and/or are involved in disease pathophysiology. Among these conditions, congenital immunodeficiency, human immunodeficiency virus (HIV) infection, autoimmune disease and HSCT have been most extensively studied.

Congenital severe combined immunodeficiency (SCID) is a heterogeneous group of severe genetic disorders of the immune system and is characterized by T cell maturation arrest with or without B cell involvement. The disease is fatal unless prompt measures are taken at very early age. Hence, early diagnosis and intervention would improve the outcome [83]. In this regard, TREC was used for the first time as a screening tool by McGhee et al. [84]. In their pilot study, dried blood spots from patients were tested for both IL7 and TREC. They reported a specificity of 100% when combining both tests and a sensitivity of 85% and 100% for IL7 and TREC respectively. Other researchers have reported results in line with this pioneering study [85,86]. Currently, TREC has proven to be the best non-invasive, feasible screening tool for SCID and has been implemented clinically in the USA. TREC analysis will most likely become a worldwide screening tool for SCID [83].

In addition to SCID, several studies have utilized TREC to assess and understand the pathophysiology of other immunodeficiency disorders such as CD4^+^ T lymphocytopenia and 22q11.2 deletion syndromes [87,88].

Monitoring TREC levels during HIV infection has caught researchers’ interest for years. Indeed, TREC was measured for the first time by Douek et al. to assess the effect of HIV on thymic activity both before and during treatment. Their results showed a decrease in thymic output in HIV-infected patients compared to age-matched healthy controls. However, after initiation of treatment and suppression of viral load, a rapid increase in TREC was observed [59].

TREC levels have also been implicated in autoimmune diseases. For instance, a marked reduction of TREC levels was shown in 51 rheumatoid arthritis patients compared to 47 healthy controls, suggesting that impaired thymic function in those patients might be a primary feature of the disease or secondary to the autoimmune process [89]. However, no reduction in TREC levels was found in another study of 70 children with juvenile idiopathic arthritis (JIA) and 110 healthy age-matched controls [90]. Finally, TREC levels were decreased significantly in patients with multiple sclerosis (MS) indicating that the thymus might play an important role in the pathogenesis of the disease [91].

The importance of TREC levels in transplantation has grown considerably. Used as a surrogate marker for thymic output, TREC has been extensively used to study different parameters that affect thymic recovery after HSCT (discussed separately in Section 2.4). It can also be employed as a predictor of immune recovery following transplantation. Furthermore, studying TREC dynamics after HSCT has significantly improved our knowledge about the mechanisms underlying T cell reconstitution.

An early study by Douek et al. [58] reported a significant correlation between TREC levels and number of naïve T cells after transplant, suggesting that TREC could predict T cell reconstitution. This finding was confirmed in other publications [57,92]. By monitoring TREC in 19 bone marrow transplantation (BMT) patients and 10 cord blood transplantation (CBT) recipients, Telvensaari et al. [93] showed that, in the long-term, higher TREC values were associated with a broader TCR repertoire and increased naïve T cell count. In addition, TREC values in CBT patients were significantly higher compared to patients transplanted with bone marrow, probably related to the very naïve characteristics of the umbilical cord blood (CB) containing hematopoietic cells.

In another study, Chen et al. [72] investigated the relationship between TREC levels prior to and after HSCT in 26 pediatric recipients. Patients with normal levels of TREC before transplantation had higher TREC levels and normal T cell count after transplantation, indicating that efficient T cell reconstitution might be predicted by the thymic status prior to transplantation.

Since rapid thymic recovery after HSCT is associated with a better outcome, TREC has also been used in transplantation for prediction of outcome. In the case of malignant hematological diseases this role is appreciated further, as T cells exhibit another level of protection through the GVL effect. In this context, several research groups including our own have studied the role of TREC as a tool to predict the rate of infection, relapse, OS and transplant related mortality (TRM) after HSCT.

For instance, results by Lewin et al. [94] have shown that low TREC levels were significantly correlated with severe opportunistic infections (OI) indicating that TREC could predict morbidity and mortality after HSCT. In the same context Clave et al. [95] measured TREC levels in preconditioning samples from 102 patients who underwent HLA-identical HSCT. Results have shown that lower TREC values before transplantation were significantly associated with increased incidence of severe bacterial and CMV infection. Also, high TREC values were associated with better survival. Similar results have been reported by Wils et al. [96] who investigated the predictive impact of TREC at different time points in a cohort of 83 HSCT patients. They showed that patients with effective recovery of TREC at 6, 9 and 12 months after transplantation had 3-, 4- and 9-fold lower risk of developing severe infections respectively. Also, TREC recovery at 6 months post transplantation could predict non-relapse mortality and OS. A study on 27 children diagnosed with hematological malignancies and transplanted with T cell depleted HLA-haploidentical grafts, showed that patients who relapsed had significantly lower sjTREC levels. Furthermore, a higher incidence of relapse was associated with lower TREC levels suggesting that assessment of TREC could predict risk of relapse in children [97]. In support, a retrospective study demonstrated that TREC levels could predict the risk of relapse in acute myeloid leukemia (AML) patients and also the survival rate in patients with chronic leukemia [69]. Sairafi et al. also reported a strong correlation between TREC levels and reduced transplant related mortality (TRM), although, in this study, a correlation between TREC level and relapse was not found [70].

In a study that included 27 patients who received double CB grafts, Brown et al. [98] studied the effect of thymic recovery on clinical outcome. The results showed that cytomegalovirus (CMV)-specific T cells could be detected earlier before TREC was detectable. However, a significant association between TREC and CMV specific T cells was reported 6 months after transplantation. Moreover, the efficiency of CMV clearance was higher in patients with TREC levels above 2000 copy/µg DNA. Furthermore, improved OS and progression free survival (PFS) was significantly associated with higher TREC levels.

TREC assessment has been used to understand the relation between thymic recovery after HSCT and GVHD. This relation has been explained in different ways. One theory is that TREC reflects the thymic ability for producing naïve recipient selected T cells that are less alloreactive. Thus, TREC levels after HSCT might predict the risk of developing GVHD. This explanation was adopted by Przybylski et al. [55] who analyzed TREC in 24 patients who underwent non-myeloablative HSCT. The results showed that patients who remained TREC negative for more than a year had developed acute GVHD grade II and III. Another explanation that will be discussed separately is that GVHD affects thymus and impairs thymopoiesis. However, Hazenberg et al. [99] proposed that low TREC levels in patients with GVHD do not necessarily indicate impaired thymic function but could be due to TREC dilution as a result of increased peripheral cell division.

### 2.4. Clinical Factors that Influence TREC Level after HSCT

As a surrogate marker for thymic output, TREC levels are affected by clinical factors that might compromise the thymus. Understanding these factors will help developing novel approaches aimed at improving thymic reconstitution. Following HSCT, thymic output and hence TREC levels are influenced by several pre- and post-transplant related factors. Of these, the most important are; recipient age, conditioning regimen, stem cell source and dose, and the occurrence of GVHD.

Age related involution of the thymus is a well-known physiologic phenomenon. Aging disrupts the microenvironment of the thymus and affects the level of essential cytokines. Many studies have shown the impact of age on thymic output. Age was negatively correlated with TREC levels before and after transplantation and positively correlated with the onset of thymic activity after HSCT, accounting for the poor prognosis in adults undergoing HSCT compared to young children [54,56,58,78,100].

The intensity of the conditioning regimen has been proposed to affect thymic output and TREC levels. Prior to HSCT, patients receive a conditioning regimen in the form of a RIC regimen or myeloablative conditioning (MAC) regimen. The role of RIC on thymic output after HSCT remains a debatable field. Studies on mice have shown that pre-transplant radiation dose affects the thymus both quantitatively and qualitatively as radiation affects thymic regenerative capacity. Furthermore, it may reduce IL-7 production, a well-known stimulus for thymic regeneration and differentiation [101]. Moreover, a recent study has shown that TBI compromises cortical and medullary thymic epithelial cells (TEC), a critical population for thymic renewal and thymopoiesis [102]. In this regard, several researchers have studied the effect of RIC on thymic reconstitution. For instance, Jimenez et al. have reported that TREC levels were significantly higher in HSCT patients receiving RIC [103]. In agreement with this study, Chen et al. reported a rapid increase in TREC and TCR diversity in patients receiving RIC [104]. In contrast with these studies, other reports have not found significant differences after transplant between RIC and MAC [100,105,106,107]. Conversely, Sairafi et al. have reported a negative correlation between TREC and RIC, however, in multivariate analysis the significance was absent [70]. It is important to point out that several reasons account for these contradictory findings; of these, the lack of randomized studies and the variations in RIC regimens protocol used are the most important [77].

The role of stem cell source and dose is elusive. Some believe that peripheral stem cell grafts contain larger numbers of stem cells and consequently should be associated with earlier T cell reconstitution and higher TREC. Conversely, others claim that graft composition is more important. In this context, studies are conflicting. For instance, in some studies, no significant correlation could be found between TREC level and stem cell source or stem cell dose [103,108]. Meanwhile, in another study, Eyrich et al. [54] reported that patients who received more than 10^7^ CD34^+^ peripheral blood stem cells (PBSC) per kg body weight had a significantly higher TREC level, although there was no positive correlation between stem cell dose and TREC level, suggesting that stem cell dose has a threshold rather than linear association. Conversely, in a univariate analysis, Clave et al. [100] have shown that TREC level was lower in patients who received PBSC graft, but they were unable to confirm this finding in a multivariate analysis. In a recent study, Sairafi et al. have shown that TREC level was higher in bone marrow (BM) graft recipients compared to PBSC graft recipients. They explained this finding by the possible beneficial role of mesenchymal stem cells and dendritic cells in the BM graft [70]. Alternatively, this reflects that BM is a more common choice for pediatric patients.

GVHD affects both thymic dependent and thymic independent T cell reconstitution. Thymic tissue is very sensitive to damage by GVHD as it is rich in antigen-presenting cells (APCs). It has been suggested that GVHD can induce damage to the thymus in different ways; one is mediated by the direct attack of allogenic immune cells, the other could be the result of using immunosuppressive drugs such as corticosteroids and cyclosporine. However, the effect of these drugs on thymocytes has only been revealed in vitro and in mice models [66]. The role of chronic GVHD in thymic tissue damage has been extensively studied. For instance, the impact of chronic GVHD on TREC level has been reported in several studies [66,92,103,108]. Few studies have reported the effect of acute GVHD on thymic output. In a multivariate analysis, Clave et al. found a strong association between acute GVHD and delayed thymic function. Interestingly, the effect of acute GVHD seems to be reversible, especially in young patients [100].

Even though recipient age, conditioning regimen, stem cell source and GVHD are the most common studied clinical factors which are thought to influence TREC levels, other factors could also potentially influence TREC levels post-HSCT. Examples of such factors are the occurrence of infections, development or sustainment of mixed chimerism and pre-transplantation thymic activity.

The occurrence of viral, bacterial and fungal infections is a severe problem for many patients undergoing HSCT and is linked to the immune reconstitution post-HSCT. It is therefore to be expected that these can affect TREC levels and vice versa. Indeed several studies have demonstrated that low TREC levels, almost immeasurable, are significantly correlated to the occurrence of severe infections in these patients [94,109].

The effect of mixed chimerism on TREC levels is unknown and very few studies have tried to elucidate the impact of it. In one study they found that pediatric patients with a prolonged mixed chimerism had faster immune reconstitution as measured by TREC levels than adult patients who reach full donor chimerism early post-HSCT [110]. However, another study found absent levels of TREC in 5 patients out of a cohort of 21 who relapsed due to mixed chimerism. Interestingly, in the same study, absent TREC levels were also found in a patient with chronic GVHD [111].

Pre-transplant thymic function is perhaps one of the least studied possible effects on TREC levels. One study used pre-transplant TREC levels as a diagnostic factor for transplant outcome [95]. They found that patients with high TREC levels have better OS, decreased incidence of severe GVHD (acute and chronic) and lower incidence of several opportunistic infections. Another study found similar results, with a correlation between high levels of sjTRECs before HSCT and a reduced risk of death mainly due to a reduction in the incidence of relapse [112]. Unfortunately, in both studies pre-transplant TREC levels were not correlated to TREC levels post HSCT or other thymic activity parameters.

All of these discussed clinical factors are unfortunately not stand-alone phenomena after HSCT. Many occur at the same time or subsequently in the same patient and may therefore influence each other as well as the TREC levels. As it is, the presence of one or more of these factors could influence the outcome of any study, thus it is difficult to have consistent conclusions among different studies. Large multivariate and multicenter analyses are needed to clarify which factors are more dominant in affecting TREC levels than others (Table 2).

## 3. Concluding Remarks and Future Role of TRECS

In recent years, numerous reports have described associations between TREC levels and variables related to treatment procedure and patient characteristics. Even though many of these analyses have included relatively large cohorts of patients, the specific results often differ from, or even contradict, those found by other investigators. One important consideration that may contribute to these inconsistencies is differences in the way TREC levels have been measured and expressed (Table 3). In some studies, TREC was calculated as a ratio between copies of signal-joint TRECs (sjTREC) and a house-keeping gene (for instance, GAPDH), measured in purified CD3^+^ cells. In other approaches, TREC levels are expressed as copies per volume of blood, or per absolute number of peripheral blood mononuclear cells (PBMCs) in a sample. The first setup seems to best improve accuracy, as the end results are not affected by variations in frequencies or concentrations of cells in peripheral blood at the time of sampling. The addition of data on ongoing rate of cell proliferation would increase the accuracy of the analysis even further, by allowing compensation for the diluting effect of peripheral expansion. It is important to reach a consensus about the method used for TREC analysis, in order to achieve results that are comparable between centers. This would enable larger multicenter trials, which would hopefully generate findings with a high level of clinical evidence.

Finally, based on the results presented here, we conclude that measurement of TREC before and after HSCT may provide clinically relevant information that can be used to evaluate patients’ current status in the process of reconstituting functional T cell immunity. This information appears to have high predictive value regarding outcome parameters, such as the risk of severe infections and survival rates. However, it is also evident that the rate and final degree of T cell reconstitution in each individual is the result of a complex interaction between thymic function and several other factors including GVHD, immunosuppression, conditioning regimen, and viral pathogens.

## Figures and Tables

**Table 1:** Alternative methods used to estimate thymic output and their limitations.

Method	Limitation
Naïve T cells (CD45RA, CD62L, CCR7)	Have longer life span, may proliferate or may convert into memory T cells also; memory cells can acquire naïve phenotype
Imaging	Provides a semi quantitative estimate of thymic output
T cell receptor excision circles (TREC)	Influenced by other factors such as the longevity of naïve T cells, peripheral expansion or apoptosis of T cells and intracellular degradation
T cell receptor diversity	Provides indirect information about thymic activity. More expensive and laborious
Novel markers such as CD31	Not all CD31^+^ naïve T cells are necessarily recent thymic emigrants (RTE). Furthermore, CD31 expression can be maintained during cytokine-driven proliferation of CD4 T cells

**Table 2:** Clinical factors influencing T cell receptor excision circles (TREC) level after hematopoietic stem cell transplantation (HSCT).

Factor	Effect on TREC Levels	Research Groups
Increasing Age	↓	Clave et al., 2009, Toubert et al., 2012, Douek et al., 2000, Eyrich et al., 2005, Politikos and Boussiotis, 2014
Acute GVHD	No effect	Jimenez et al., 2006
	↓	Clave et al., 2009
Chronic GVHD	↓	Eyrich et al., 2005, Jimenez et al., 2006, Jimenez et al., 2005, Weinberg et al., 2001, Fallen et al., 2003
Reduced-intensity conditioning (RIC)	↑	Chen et al., 2006, Jimenez et al., 2005
	No effect	Maris et al., 2003, Morecki et al., 2001, Clave et al., 2009, Geyer et al., 2011
	↓	Sairafi et al., 2012
Peripheral blood stem cells as graft source	↓ No effect	Clave et al., 2009, Sairafi et al., 2012 Fallen et al., 2003, Jimenez et al., 2005
Cyotmegalovirus infection	↓	Jimenez et al., 2006

An arrow up denotes a positive effect of a factor on TREC levels. An arrow downwards denotes a negative effect of a factor on TREC levels.

**Table 3:** Variabilities in the methods used for T cell receptor excision circles (TREC) estimation.

TREC Methods	Research Groups
Detection method	
Semi quantitative PCR	Douek et al., 1998, Douek et al., 2000
Hybridization probe	Jimenez et al., 2006, Loeffler et al., 2002
Molecular beacon	Zhang et al., 1999
TaqMan probe	Hazenberg et al., 2000, Przybylski et al., 2007, Mensen et al., 2013, Lorenzi et al., 2008, Sugita et al., 2008, Sairafi et al., 2012
SYBR Green PCR	Ponchel et al., 2003
PCR-ELIZA	Al-Harthi et al., 2000
Cell population	
CD3	Ringhoffer et al., 2013, Jimenez et al., 2006, Sairafi
CD4/CD8	Douek et al., 1998, Fallen et al., 2003, Sugita et al., 2008
Peripheral blood mononuclear cells	Przybylski et al., 2007, Douek et al., 2000, Eyrich et al., 2005
Type of TREC	
sj TREC	The majority
sjTREC/βTREC	Dion et al., 2004, Ringhoffer et al., 2013, Ferrando-Martinez et al., 2010
Result expression	
TREC/cell count	Przybylski et al., 2007
TREC/mL or μL of Blood	Lorenzi et al., 2008, Eyrich et al., 2005, Chen et al., 2005
TREC/μg DNA	Jimenez et al., 2006, Douek et al., 2000
Absolute TREC count	The majority
Relative (Delta *C*_t_ method)	Uzunel et al., 2014, Sairafi et al., 2012
